# Mesenchymal Stromal Cell Therapy in Solid Organ Transplantation

**DOI:** 10.3389/fimmu.2020.618243

**Published:** 2021-02-10

**Authors:** Manuel Alfredo Podestà, Giuseppe Remuzzi, Federica Casiraghi

**Affiliations:** ^1^ Department of Health Sciences, Università degli Studi di Milano, Milan, Italy; ^2^ Istituto di Ricerche Farmacologiche Mario Negri IRCCS, Aldo & Cele Daccò Clinical Research Center for Rare Diseases, Bergamo, Italy

**Keywords:** mesenchymal stromal cells, regulatory cells, tolerance, kidney, liver, lung

## Abstract

Transplantation is the gold-standard treatment for the failure of several solid organs, including the kidneys, liver, heart, lung and small bowel. The use of tailored immunosuppressive agents has improved graft and patient survival remarkably in early post-transplant stages, but long-term outcomes are frequently unsatisfactory due to the development of chronic graft rejection, which ultimately leads to transplant failure. Moreover, prolonged immunosuppression entails severe side effects that severely impact patient survival and quality of life. The achievement of tolerance, i.e., stable graft function without the need for immunosuppression, is considered the Holy Grail of the field of solid organ transplantation. However, spontaneous tolerance in solid allograft recipients is a rare and unpredictable event. Several strategies that include peri-transplant administration of non-hematopoietic immunomodulatory cells can safely and effectively induce tolerance in pre-clinical models of solid organ transplantation. Mesenchymal stromal cells (MSC), non-hematopoietic cells that can be obtained from several adult and fetal tissues, are among the most promising candidates. In this review, we will focus on current pre-clinical evidence of the immunomodulatory effect of MSC in solid organ transplantation, and discuss the available evidence of their safety and efficacy in clinical trials.

## Introduction

Solid organ transplantation has been established as the standard of care for end-stage disorders affecting the kidneys, liver, heart, lungs and small bowel. Advances in our understanding of the adaptive host-versus-graft immune response have led to the development of potent immunosuppressive agents that have improved graft and patient survival in early post-transplant stages substantially (1). Despite these breakthroughs, the current immunosuppressive regimen is associated with detrimental side effects, such as cardiovascular diseases (2), metabolic complications (3), cancer (4), and infections (5), which cause significant morbidity and mortality. Moreover, immunosuppressants are ineffective in preventing the development of chronic rejection, which causes 10% of kidney allograft loss every year (6) and affects 50% and 75% of lung transplant recipients at 5 and 10 years post-transplant (7). Therefore, there is an urgent need for alternative strategies to enable the minimization of immunosuppression and to improve long-term graft survival. Among these, the use of live suppressor/regulatory cells is emerging as the most promising tool. The use of mesenchymal stromal cells (MSC) is gaining particular attention due to their potential to inhibit the host-versus-graft immune response at the different key steps involved in acute and chronic graft rejection.

In this review we provide a summary of the immunomodulatory features of MSC in pre-clinical models of solid organ transplantation and analyze the results of clinical studies using MSC-based cell therapies in patients with kidney, liver, lung, and small bowel transplantation.

### Mesenchymal Stromal Cells

Mesenchymal stromal cells are plastic-adherent, non-hematopoietic, fibroblast-like cells with the capability to differentiate into osteoblasts, adipocytes and chondrocytes. Traditionally, bone marrow (BM) was the main source of MSC considered; however, alternative sources, such as adipose tissue (8), the umbilical cord (9), or the placenta (10) are now widely used as sources of MSC due to their higher yield and the less invasive procurement strategies involved.

MSC are isolated and expanded in culture from whole cell preparations by using their ability to adhere to cell culture plastic and to proliferate for several weeks. This approach yields a population of fibroblast-like cells that are relatively homogenous morphologically, but it inevitably contains a heterogeneous population of cells with distinct phenotypes and biological properties. In 2006, the International Society for Cell & Gene Therapy established a non-ambiguous and broadly accepted set of minimal criteria for defining “mesenchymal stromal cells”: plastic-adherence, expression of CD105, CD90, and CD73 surface markers, negativity for CD45, CD19, and CD14 hematopoietic antigens, and stimulus-induced tri-lineage differentiation *in vitro* into osteoblasts, adipocytes and chondrocytes (11).

The lack of specific markers and the retrospective characterization of MSC (which is still performed after long-term culture) have long precluded a deeper understanding of their native origin and physiological functions (12). Studies conducted during the last decades showed that MSC represent a fundamental component of the BM stroma, where they control maintenance, self-renewal and differentiation of hematopoietic stem cells (12, 13). Impaired functional, replicative, and regenerative capacities of BM-MSC have been implicated in development of hematological malignancies (14), such as myelodysplastic syndromes (15, 16), leukemia (17), and multiple myeloma as well as in BM failure syndromes (18, 19). More recent evidence suggests that MSC reside in the vascular niches, being either identical to or deriving from pericytes (20). Here, MSC stabilize the vascular network, contribute to the normal tissues and immune homeostasis, and modulate osteoclast formation. In response to injury, MSC participate in tissue repair and might inhibit overaggressive autoimmune reaction against the injured tissue (21).

Despite arguments about heterogeneity and *in vivo* counterparts (22), a wealth of data has provided irrefutable evidence that MSC have unique and highly potent immune-dampening, immune-regulatory, anti-inflammatory, and pro-reparative properties. This evidence, coupled with simple and cost-effective cell production, have stimulated intense investigation of MSC as a novel therapy for numerous clinical indications (23), including solid organ transplantation (24).

### Immunomodulatory Features of MSC on Adaptive Immunity

One of the first pieces of evidence of the immunomodulatory effect of MSC was provided—almost 20 years ago—in a baboon skin allograft model (25). In this study, MSC were shown to suppress allogeneic T-cell proliferation in a mixed lymphocyte reaction and to delay skin allograft rejection (25). Since then, numerous *in vitro* and *in vivo* studies have demonstrated the capability of MSC to inhibit the activation and proliferation of CD4^+^ T cells (26), preventing their differentiation into T_H_1 and T_H_17 effector cells (27), and to reduce CD8^+^ T-cell cytotoxicity in response to allogeneic stimuli (28). MSC were shown to also suppress the activation of memory T cells induced by cytokines (29) or by alloantigens from both minor and major histocompatibility complexes (30, 31).

Of particular interest, MSC exhibited a remarkably potent ability to convert not only naïve (32, 33) but also effector/memory CD4^+^ T (34, 35) and CD8^+^ T cells (36–38) toward a regulatory phenotype. Indeed, in *in vitro* studies, human BM-MSC expanded Tregs from CD3^+^CD45RO^+^ human memory T cells (34) and from collagen-reactive human T cells, including CD8^+^ T cells (36, 39). MSC-induced CD4^+^ Tregs maintained a regulatory phenotype and function over time (34) and suppressed the *ex vivo* proliferation of T cells from patients with rheumatoid arthritis in an antigen-specific manner (39). The mechanisms at the basis of this Treg-inducing capacity are incompletely understood, but likely involve cell-to-cell contact (40, 41), the release of soluble mediators such as Transforming Growth Factor (TGF)-β1 (40, 41) and Prostaglandin E2 (PGE2) (41), as well as the induction of regulatory phenotype in antigen presenting cells (37, 41). PGE2 (35) and Hepatocyte Growth Factor (HGF) (42) had a key role in the induction of a Treg phenotype in differentiated Th17 cells, either after *in vitro* polarization (42) or isolated from inflamed tissues from patients with psoriasis vulgaris or active Crohn’s disease (35). A very recent study described that the transfer of mitochondria from MSC to CD4^+^ T cells may be a mechanism capable of driving Treg differentiation by itself (43).

Regardless of the underlying mechanisms, Treg induction by MSC has so far been observed consistently in several animal models of immunological diseases (41), in different human autoimmune disease conditions (44–46), as well as in acute and chronic graft-versus-host disease (GVHD) (47, 48).

Recent studies in autoimmune disorders have identified follicular T helper cells (T_FH_) as an additional target of MSC immunomodulation. MSC downregulated the proliferation and differentiation of T_FH_ cells during *in vitro* polarizing conditioning of CD4^+^ T cells isolated from patients with rheumatoid arthritis (49) and Sjogren syndrome (50), or from lupus-prone mice (51–53). Indoleamine 2,3-dioxygenase (IDO) (49) and inducible Nitric Oxide Synthase (iNOS) (52) expression or cell-contact (51) have been reported as possible mechanisms. MSC infusion in NZB/W (51) or MRL/lpr (52, 53) lupus-prone mice or in mice with collagen-induced arthritis (CIA) (49) attenuated disease severity, reduced autoantibody levels and was associated with a decrease in the frequency of T_FH_ cells. A very recent study in a mouse model of chronic GVHD (54) showed that extracellular vesicles isolated from human umbilical cord (UC)-MSC alleviated disease manifestation by reducing germinal center B cell and T_FH_ cell number in the spleen (54). Moreover, T_FH_ cells isolated from UC-MSC treated CIA mice inhibited *ex vivo* proliferation, differentiation and IgG production from B cells (49). This evidence suggests that MSC could indirectly regulated the B cell responses in autoimmune diseases by exerting their inhibitory action on T_FH_ cells.

Conflicting results have been reported on the direct effects of MSC on B cells. Some groups found that MSC may inhibit *in vitro* proliferation of B cells and their differentiation into plasma cells (55), while other authors described an opposite effect (56). These discrepancies can be explained by the different experimental conditions used in these studies, such as the starting B cell population—whether purified B cells or total lymphocytes—as well as the type of stimuli used for activating B cells, and the effects these stimuli could directly exert on MSC (57). Nevertheless, recent findings indicate that MSC inhibit proliferation of and IgG production of B cells in the presence of activated by T cells (58) or inflammatory cytokines (59, 60). In contrast, direct interaction between MSC and B cells mainly affected B cell differentiation, resulting in reduced plasmablast formation and increased generation of IL-10–secreting regulatory B cells (59, 60).

The generation of Bregs by MSC have been confirmed in *in vivo* studies. In mouse models of multiple sclerosis (61) and lupus (62), treatment with MSC suppressed the severity of the disease by increasing the frequency and activity of Bregs along with an enhanced secretion of IL-10. In a clinical study, patients with refractory chronic GVHD given MSC infusions had clinical improvements associated with increased proliferation and IL-10 production by Bregs (63).

Overall, these studies indicate that MSC have a broad immunomodulatory actions on cells of the adaptive immune system, modulating effector functions and promoting regulatory properties.

### Immunomodulatory Features of MSC on Innate Immunity

Another fundamental immunoregulatory property of MSC is their effect on antigen-presenting cells.

In *in vitro* experiments, MSC impaired dendritic cell maturation, downregulating their expression of MHC-II and costimulatory molecules (64, 65) and preventing the secretion of the pro-inflammatory cytokines IL-12, IFNγ, and TNFα (66). Consequently, DC exposed to MSC exhibited impaired alloantigen presentation and inefficient effector T-cell activation (65). These effects, coupled with enhanced secretion of the anti-inflammatory cytokine IL-10, resulted in sustained expansion of regulatory T cells (Tregs) (67). In addition, MSC inhibited *in vivo* DC migration toward lymphoid organs by downregulating CCR7 expression (65).

Among the cells of the innate immune system, macrophages are the main target of MSC immunoregulation, as highlighted by several recent studies. MSC promote macrophage polarization toward the anti-inflammatory M2 phenotype (68), downregulating the secretion of pro-inflammatory cytokines while upregulating phagocytic activities and the release of IL-10 (69). MSC, either by inducing (70) or undergoing (71) apoptosis, enable macrophages to produce TGFβ and to promote the induction of Tregs. In addition, by releasing trophic factors, MSC play an important role in educating macrophages to promote tissue repair and inflammation resolution (72).

The mechanisms through which MSC exert these effects on the multiple adaptive and innate immune effector cells are incompletely understood. However, paracrine effects mediated by their plentiful secretome, which includes cytokines, growth factor, and miRNA directly transferred to close target immune cells or encapsulated in extracellular vesicles, appear to be among the main mechanisms of MSC immunomodulation. Key mediators include TGFβ (73); HGF (39); PGE2 (69); IDO (74); iNOS (75); leukemia inhibitor factor (LIF) (76); HLA-G1 (77); TNF-stimulated gene 6 (TSG-6) (78); galectin-1, -3 and -9 (79); purinergic signals (80), as well as miRNA targeting TLR-associated pathways and the inflammasome (81) and mitochondrial transfer (82).

Overall, it is now clear that it would be impossible to identify a single mechanism responsible for the effect of MSC: different mediators released by MSC or surface molecules expressed on these cells are likely to act in concert to inhibit the alloimmune response at several crucial points, inducing the differentiation and proliferation of Tregs, Bregs and immature DC and M2 macrophages to dominate the anti-graft immune response. The establishment of a regulatory cell network could resolve the long-standing conundrum of the long-term effects of MSC in spite of their very short-term engraftment and *in vivo* survival (83).

### Insights From Experimental Models of Solid Organ Transplantation

**Figure 1:**
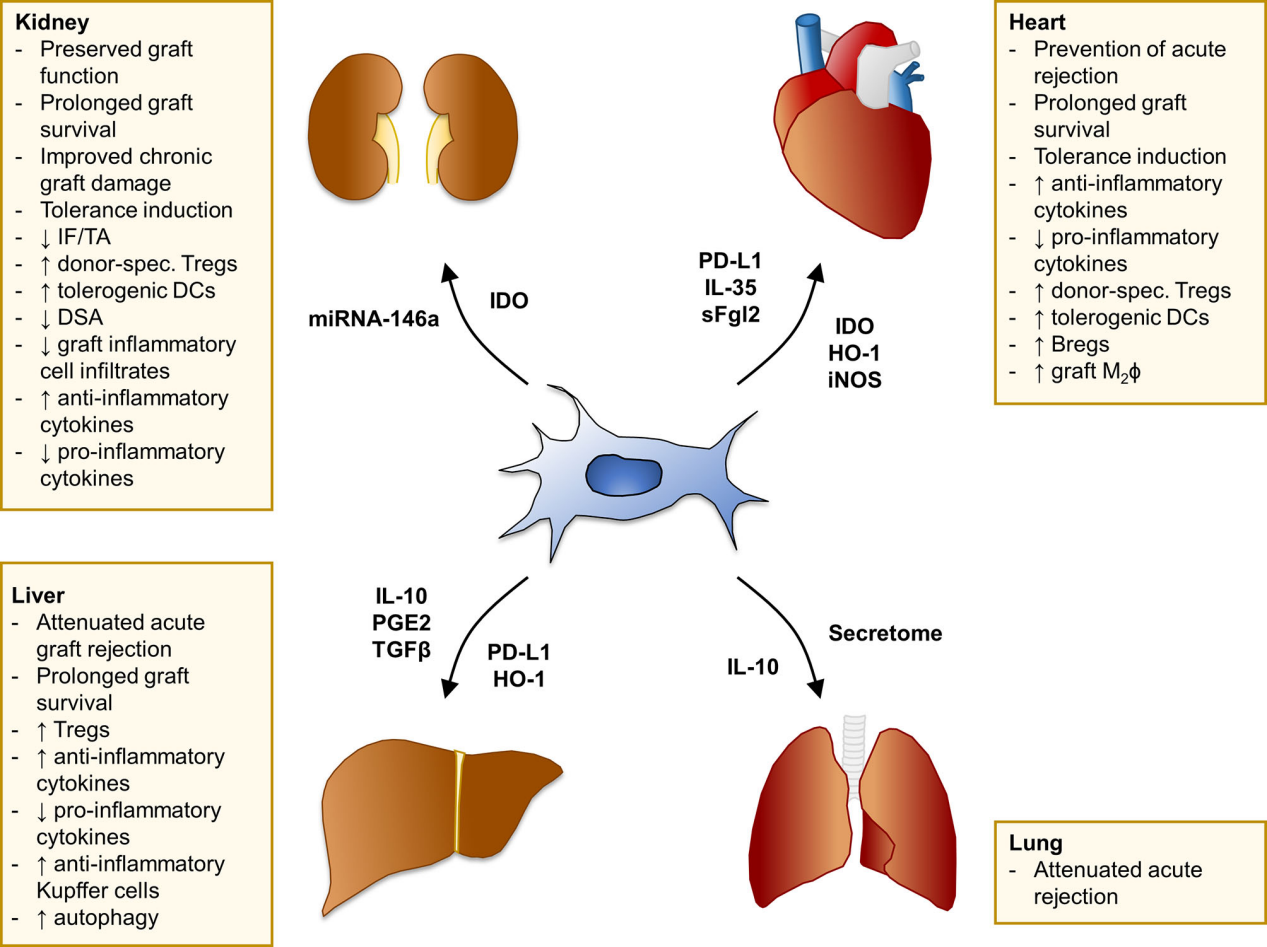
Summary of MSC effects in pre-clinical models of solid organ transplantation. Main findings of studies with MSC in experimental models of kidney, heart, liver, and lung transplantation. The mediators involved in MSC-induced pro-tolerogenic effects and/or specifically overexpressed in selected MSC cell-lines through genetic engineering are listed next to each arrow. Bregs, regulatory B cells; DCs, dendritic cells; DSA, donor-specific antibodies; HO-1, heme oxygenase-1; IDO, indoleamine 2,3-dioxygenase; IF/TA, interstitial fibrosis/tubular atrophy; IL-, interleukin-; iNOS, inducible nitric oxide synthase; M_2_ϕ, M2 macrophages; PD-L1, programmed death-ligand 1; PGE2, prostaglandin E2; sFgl2, soluble fibrinogen-like protein 2; TGFβ, transforming growth factor β; Tregs, regulatory T cells.

MSC have been the subject of vigorous investigation as a potential tolerogenic cell therapy in pre-clinical transplant models of the kidney, heart, liver and lung (**Figure 1**).

#### Kidney Transplantation

In murine models of acute transplant rejection following kidney transplantation, an intravenous injection of MSC derived from either donor mice (84) or syngeneic recipient mice (85, 86) induced graft tolerance, mediated by the generation of donor-specific FOXP3^+^ Tregs (84–86) and tolerogenic dendritic cells (84, 87). The main mediator involved appeared to be IDO, since MSC from IDO knock-out mice failed to prolong graft survival (84). These findings have been confirmed in a rabbit model of kidney transplantation, where the induction of donor-specific Tregs and graft tolerance mediated by bone marrow-derived MSC (BM-MSC) were strengthened by transgenic IDO overexpression (88). The Treg-inducing property was found to be dependent on MSC localization in secondary lymphoid organs, before (85) or at the beginning (86) of the immune response, since MSC injection 2 days after transplant failed to expand Tregs and to induce long-term graft acceptance (85, 86).

Similarly, the administration of BM-MSC as multiple (89) or single (90) intravenous injections in rats undergoing kidney transplantation preserved renal function in the early post-transplant and reduced graft mRNA levels of inflammatory cytokines and the number of infiltrating macrophages and dendritic cells, while increasing graft FOXP3^+^ Tregs. These positive effects increased when BM-MSC were induced to overexpress CXCR4, a procedure that upregulated the MSC expression of anti-inflammatory factors (90).

MSC have also shown the potential to improve chronic kidney graft damage (91–94). In rat models of chronic graft injury, the administration of BM-MSC at both early (91, 93, 94) and late (92) post-transplant time points reduced T-cell and macrophage graft infiltration, inhibited the mRNA expression of inflammatory cytokines and prevented the development of interstitial fibrosis, tubular atrophy and glomerulosclerosis, as well as of donor-specific antibodies (91–94).

Despite these very promising results, a number of reports have described severe complications following MSC infusion, raising concerns about the safety of this cell therapy. We observed that MSC infusion 2 days after kidney transplantation was associated with transient graft dysfunction characterized by increased complement C3 deposition and neutrophil infiltration. In rat kidney transplant models, the injection of MSC from either syngeneic bone marrow (95) or donor adipose tissue (96) was associated with increased mortality of recipient rats due to thrombotic microangiopathy, renal infarctions and infection (95) or to premature graft loss (96).

#### Heart Transplantation

In heterotopic heart transplant models, the administration of MSC from bone marrow or adipose tissue, either of donor or recipient origin, mildly but significantly prolonged heart graft survival (97–100). The beneficial effect of MSC on graft survival prolongation translated into long-term graft acceptance when cell infusion was associated with a short post-transplant course of mycophenolate mofetil (100, 101) or rapamycin (99, 102). The infusion of MSC before transplantation achieved better results than post-transplant administration, and the maintenance of MSC-mediated tolerance was noticeably dependent on the generation of donor-specific regulatory T cells (99, 102), as we first demonstrated in a semi-allogeneic heart transplant model (103). The transfection of MSC with IL-35 (104) or soluble fibrinogen-like protein 2 (105), two molecules involved in Treg generation and function, was able to boost the immunomodulatory effect of MSC in preventing acute rejection. The increase in Treg cells following MSC administration was also associated with the development of tolerogenic DC (99, 102) and regulatory B cells, effects that were mediated mainly by programmed death-ligand 1 (PD-L1) expression (102) and an increased proportion of graft M2 macrophages (105).

#### Liver Transplantation

MSC therapy has been tested extensively in pre-clinical liver transplant models. All of the studies demonstrated that MSC, isolated from the bone marrow of both donor and recipient origin (106, 107), as well as from adipose tissue (108, 109), and injected on the day of transplantation, can attenuate acute graft rejection, prolong graft survival, inhibit T_H_1 activation and reduce the release of pro-inflammatory cytokines while promoting anti-inflammatory cytokine generation and the emergence of FOXP3 regulatory T cells (106, 107, 109). MSC were also found to be effective in a large animal model (108), and in small-for-size (109) and non-heart beating donor (110) liver transplant models.

The Treg-generating ability of MSC was also beneficial in preventing—but not in reversing—the development of post-liver transplant acute GVHD (111). Indeed, the administration of either donor-derived or syngeneic BM-MSC into LEWxBNF1 recipients during the first 7 days after LEW liver transplant prevented the onset of acute GVHD mediated by LEW splenocytes injected post-operatively. The injection of MSC between 8 and 14 days after transplantation failed to reverse GVHD symptoms, suggesting, also in this setting, the importance of the timing of cell administration in order to fully take advantage of MSC immunomodulation (111).

In the setting of liver transplantation, different key immunomodulatory molecules have been overexpressed in MSC to enhance their tolerogenic properties and improve liver transplant outcomes. MSC overexpressing IL-10 (112), PGE2 (113), TGFβ (114), and HO-1 (115, 116) increased the capability to skew the Treg/T_H_17 balance (112, 116), to promote the development of induced-Tregs (2) and to convert Kupffer cells toward an anti-inflammatory phenotype (113). HO-1 overexpression conferred a higher cytoprotective effect on MSC by promoting autophagy (117) and by improving hepatic sinusoidal microcirculation and energy metabolism (118). Notably, MSC transfected with PDL1-Ig were found to induce long-term graft tolerance in a rat model of liver allotransplantation (119).

#### Lung Transplantation

In rat models of orthotopic left lung transplantation, human BM-MSC given as a double injection of 3 × 10^6^ cells *via* the left pulmonary artery at day 0 and intravenously at day 3 post-transplantation decreased lymphocytic infiltrates, edema and hemorrhage at the histological examination 6 days after transplant, even though the total acute rejection score was reduced only mildly (120). A more remarkable effect was achieved when MSC were associated with conventional immunosuppression. The co-administration of MSC isolated from autologous adipose tissue with tacrolimus significantly reduced rejection scores at day 7 post-transplantation, and this effect was associated with a reduced frequency of proliferating cell nuclear antigen (PCNA)-positive cells in bronchus-associated lymphoid tissue cells (121), suggesting that MSC could inhibit the local early rejection process (122).

Similarly, the administration of IL-10 overexpressing-BM-MSC together with CsA improved graft function and alleviated 5-day acute rejection (123), an effect that could be reproduced through the use of daily intratracheal injections of conditioned media from unmanipulated BM-MSC (124), suggesting the MSC secretome plays a major role in inhibiting the early phase of acute lung allograft rejection.

Overall, these studies in pre-clinical transplant models have demonstrated that MSC have a powerful capacity to skew the host-versus-graft immune response toward a regulatory phenotype, promoting a pro-tolerogenic environment dominated by donor-specific Tregs (**Figure 1**). How MSC, regardless of their origin (i.e., autologous, donor- or third party-derived) can promote the expansion of donor-specific Tregs and the development of tolerance is not completely understood. Several studies showed that MSC potently induce Tregs (41), mainly by converting conventional T cells into Tregs (33, 40). This likely results in the expansion of a broad repertoire of polyclonal T cells with different specificities. The leading hypothesis is that the antigen pressure deriving from the graft could lead to the selection of Tregs able to recognize donor antigen, therefore receiving the correct TCR signaling for survival advantage and long-term dominance. Moreover, MSC can sense the microenvironment and, depending on the prevailing immunological milieu they encounter *in vivo*, may modulate both their phenotype and the function of immune cells from the host. The timing of cell infusion and the degree of T-cell activation are the most crucial factors in determining the beneficial effect of MSC in the transplant setting. Highly activated T cells and an inflammatory environment can hamper MSC-mediated immunosuppression or even promote their conversion into pro-inflammatory cells.

### Clinical Studies

#### Kidney Transplantation

After encouraging results were obtained in animal models and following reports of the efficacy of MSC in treating graft-versus-host disease in bone marrow transplant recipients (125), our group was the first to translate MSC therapy to clinical trials in solid organ transplantation (126). Since then, several research groups have tried to determine the extent of the immunomodulatory effects that MSC have in clinical settings. In renal transplantation, MSC have been used with different aims: to induce operational tolerance to the allograft, to treat subclinical rejection, thus preventing the development of chronic tissue damage and renal function deterioration, or to reduce the overall dose of induction and/or maintenance immunosuppression.

In the pursuit of immune tolerance, our group designed a phase 1 clinical study to assess the safety and feasibility of MSC administration in two recipients of living-donor kidney transplants, whose preliminary results were first reported over ten years ago. Autologous BM-MSC at a dose of 1 to 2 × 10^6^ cells/kg were infused seven days after transplantation, following induction with low-dose anti-thymocyte globulins and basiliximab (126). Immune monitoring revealed a progressive increase in the Treg fraction and a marked reduction in the percentage of circulating CD8^+^ memory T cells, coupled with reduced donor-specific T-cell alloreactivity. However, due to the occurrence of transient renal dysfunction without evidence of rejection in both patients, the MSC infusion schedule was reconsidered; indeed, post-transplant MSC administration was shown to be associated with MSC intra-graft migration and pro-inflammatory polarization, resulting in severe neutrophilic infiltration and C3 deposition. Consistent with studies in animal models (85, 127), this engraftment syndrome was completely abrogated by infusing MSC the day before renal transplantation (128).

Long-term follow-up highlighted a sustained increase in the ratio between Treg and CD8^+^ effector T cells in one of these patients, which was associated with a B-cell profile consistent with the pro-tolerogenic signature identified in other cohorts of spontaneously tolerant kidney transplant recipients (129). This patient consented to gradual tapering of immunosuppression, which was successfully completed without any evidence of rejection (the patient has been off immunosuppression for over two years), thus supporting the hypothesis that a single administration of MSC may induce a long-term, self-sustaining immunoregulatory process responsible for tolerance induction (130). Other groups reported similar immunomodulating effects after the administration of autologous BM-MSC, which were safe and induced an increase in Treg frequency and a reduction in T-cell proliferation (131); nevertheless, so far immunosuppression withdrawal has not been attempted in any other study on renal transplant recipients.

Delayed administration (i.e., over 4 weeks and up to 6 months after transplantation) of autologous BM-derived MSC was instead used by Reinders and colleagues to treat patients who exhibited signs of subclinical rejection or interstitial fibrosis/tubular atrophy on protocol biopsies (132). Most of these recipients displayed donor-specific hypo-responsiveness in T-cell proliferation assays, and the resolution of tubulitis was reported in the two patients who underwent repeat renal biopsy.

Several investigators also exploited MSC immunomodulation to safely reduce, but not completely withdraw, induction and/or maintenance immunosuppression. The efficacy of peri- and post-transplant infusion of autologous BM-derived MSC as a replacement of induction therapy with basiliximab was assessed in a randomized controlled trial involving 159 patients (133). Independent of the maintenance immunosuppression dose, patients allocated to MSC had a significantly lower incidence of acute rejection and renal function decline.

Similar results were obtained with the use of allogeneic, donor-derived MSC, which reportedly allowed a 50% dose reduction of calcineurin inhibitors without having an impact on the incidence of rejection episodes, graft function or survival (134, 135). Despite these results, these studies did not find any difference in the immunophenotype of MSC recipients over time, underscoring that a certain degree of variability in results due to the heterogeneity of MSC preparations, timing of infusion, concomitant immunosuppression and patient selection needs to always be considered in these trials.

These initial experiences with non-autologous MSC paved the way for the use of off-the-shelf third-party allogeneic MSC, which have the invaluable advantage of prompt availability for use in deceased-donor renal transplantation. Sun and colleagues first reported that pre-transplant infusion of third-party umbilical cord-derived MSC (UC-MSC) under standard immunosuppressive therapy (including anti-thymocyte globulins) was safe and well tolerated in deceased-donor renal transplant recipients (136).

Early post-transplant administration of third-party BM-MSC obtained consistent results, and immunophenotype monitoring showed increased frequency of Treg compared to the control group (137). However, the same study also indicated that 40% of patients developed *de novo* donor-specific antibodies (DSA) against MSC or shared graft-MSC HLA, whose long-term relevance is still largely unknown.

Dreyer and colleagues recently reported the results of a clinical trial assessing the safety of a single third-party BM-MSC infusion 6 months after transplantation with a concomitant reduction of maintenance immunosuppression (138). To reduce the risk of sensitization against graft-relevant antigens, the investigators designed an allocation strategy to avoid repeated mismatches between the graft and the MSC product. At variance with the aforementioned study, none of the patients developed *de novo* DSA, possibly due to the more quiescent immunologic state at the time of MSC infusion compared to the peri-transplant period. Notably, no significant change in leukocyte subsets was observed after MSC infusion, suggesting that delayed administration may have limited immunomodulatory effects in this setting.

#### Liver Transplantation

Similarly to renal transplantation, MSC immunomodulatory properties were exploited for heterogeneous purposes in liver graft recipients, including the induction of operational tolerance, inhibition of acute rejection and treatment of ischemic biliary lesions.

The safety and feasibility of early post-transplant infusion of third-party BM-MSC (1.5–3.0 × 10^6^ cells/kg) was assessed in ten liver transplant recipients participating in a controlled, open-label, non-randomized clinical trial (139). Within the limits of the short follow-up, MSC did not increase the risk of infection or malignancy, and the rates of graft rejection, survival and histologic analysis of 6-month protocol biopsies were similar between patients who received MSC and controls. However, MSC failed to induce changes in the immunophenotype, and weaning MSC recipients off immunosuppression was not successful.

Intriguing results were reported with the use of MSC for the treatment of biopsy-proven acute liver allograft rejection. Twenty-seven patients were randomly allocated to receive conventional immunosuppression with or without UC-MSC infusion. At the end of the 12-week follow-up, the patients who received MSC exhibited lower liver enzyme levels, increased frequency or circulating Treg and improved histology compared to controls (140).

The therapeutic potential of six doses of UC-MSC (1.0 × 10^6^/kg each) was also assessed in 12 liver transplant recipients with ischemic-type biliary lesions (141). Compared to a group of patients treated with a traditional protocol, those who received MSC had a significantly lower need for interventional therapeutic procedures, lower mortality and higher graft survival.

#### Lung Transplantation

In lung transplant recipients the use of MSC has focused on treating chronic allograft dysfunction, the main limitation to long-term graft and patient survival in this setting. A single-arm, phase 1 trial assessed the safety and feasibility of four infusions of allogeneic third-party BM-MSC (2 × 10^6^ cells/kg) in 10 patients with progressive chronic lung allograft dysfunction (142). The therapy was well tolerated, and no adverse events involving hemodynamics or gas exchanges were reported. The authors observed a trend toward a slower rate of decline in forced expiratory volume in one second in MSC-treated patients. Nonetheless, two patients died during follow-up due to progressive graft dysfunction, suggesting that the effect of MSC may be heterogeneous in this context as well.

The therapeutic potential of a single infusion of third-party BM-MSC was also assessed by Keller and colleagues in a dose-escalation trial that enrolled a relatively homogenous cohort of 9 patients with moderate chronic lung allograft dysfunction (143, 144). Gas exchanges and pulmonary function tests did not change significantly immediately after infusion or during the first month of follow-up. However, lung function parameters stabilized after MSC infusion and did not significantly decline at one year of follow-up, a finding consistent with a possible beneficial effect of MSC on the progression of chronic lung allograft dysfunction.

#### Small Bowel Transplantation

The properties of MSC have also been assessed in a few cases of small bowel transplantation. A preliminary report described the case of an HLA-matched small bowel graft recipient who developed severe refractory bowel dysfunction (145). The patient was treated with a single infusion of allogeneic BM-MSC (1 × 10^6^ cells/kg) as rescue therapy with the dual intent of providing immunosuppression and support for tissue regeneration. An early, marked functional and histological improvement was noted in the first two weeks after treatment, and the patient remained stable up until 2 months of follow-up.

Peri- and post-transplant intra-graft administrations of autologous BM-MSC (three doses, 1 × 10^6^ cells/kg each) were also employed in a case series of 6 patients who underwent small bowel transplantation (146). Half of these patients experienced severe acute rejection, an event rate that is similar to other patient series of small bowel transplantation (147), and died due to complications within 3 months of surgery. The results of this study indicate that MSC are safe in small bowel transplantation as well, but the small number of patients treated so far mandates further studies before definitive conclusions on their effects can be drawn.

## Open Issues and Future Perspectives

Pre-clinical studies have clearly demonstrated the potential of MSC to substantially improve outcomes in solid organ transplantation. On the other hand, the clinical studies conducted so far were mainly phase 1 trials, which were designed to assess the feasibility and safety of MSC therapy.

In our opinion, original concerns regarding a potentially higher risk of infections and malignancy in MSC recipients have been progressively debunked by these trials. Indeed, one of the first studies in kidney transplant recipients raised the issue of increased incidence of opportunistic infections in patients who received MSC (132), but these results were not confirmed by other studies (133, 137, 148). Similarly, human MSC did not demonstrate any potential of malignant transformation, even after long-term *in vitro* expansion, and no association between MSC and cancer has been reported in any of the trials conducted so far (149, 150). Overall, this provides a strong signal regarding MSC safety, even in this context, but long-term surveillance still needs to be implemented, as most of these trials reported results during a limited follow-up period.

Despite the inherent design limitations of phase I studies, MSC have shown some degree of efficacy in protecting the graft from chronic rejection and in promoting a pro-tolerogenic environment, even in this setting. Nonetheless, these effects are not as robust as those demonstrated in pre-clinical studies.

Several factors are at the basis of the limited success of MSC therapy in humans. First, despite decades of intense research, the precise mechanism through which MSC interact with the host immune system has not been completely understood yet. An improved understanding of the mechanism of action of MSC will be crucial in allowing the set-up of assays for selecting the most effective cell preparation *a priori*, in enabling the standardization of cell manufacturing processes in cell factories, and in establishing the appropriate dose, timing, source and concomitant immunosuppressive therapy to favor the beneficial effects of MSC. Identifying the most important mediator(s) of MSC-induced immunomodulation will also make it possible to clarify whether engineering MSC could provide additional benefits *in vivo* compared to standard preparations, or whether MSC secretome could replace live cells for cell-free tolerogenic therapy.

Research should also aim to develop methods to identify biomarkers of response to MSC therapy in transplant patients. This will make it possible to identify factors that can influence MSC therapeutic efficacy *in vivo*, such as recipient age, medical history, underlying diseases and type of solid organ transplant. These factors would enable the selection of candidates who would benefit from MSC therapy and the tailoring of MSC therapy to each solid organ transplant recipient.

Once these outstanding challenges are addressed adequately, we might finally be able to make a major breakthrough in the induction of tolerance to solid organ transplantation.

## Author Contributions

FC and MP searched the literature and wrote the first draft of the manuscript. GR critically revised the work. All authors contributed to the article and approved the submitted version.

## Funding

This work was partially supported by the Agenzia Italiana del Farmaco (AIFA, Rome, Italy; project MESNEPH), and by the European Commission (Horizon 2020, project NEPHSTROM). The materials presented and views expressed here are the responsibility of the authors only. Funding bodies take no responsibility for any use made of the information set out.

## Conflict of Interest

The authors declare that the research was conducted in the absence of any commercial or financial relationships that could be construed as a potential conflict of interest.
